# Zn tolerance in the evergreen shrub, *Aucuba japonica*, naturally growing at a mine site: Cell wall immobilization, aucubin production, and Zn adsorption on fungal mycelia

**DOI:** 10.1371/journal.pone.0257690

**Published:** 2021-09-30

**Authors:** Kohei Doyama, Keiko Yamaji, Toshikatsu Haruma, Atsushi Ishida, Shigeta Mori, Yoko Kurosawa

**Affiliations:** 1 Graduate School of Life and Environmental Sciences, University of Tsukuba, Tsukuba, Ibaraki, Japan; 2 Center for Ecological Research, Kyoto University, Otsu, Shiga, Japan; 3 Department of Agriculture, Yamagata University, Tsuruoka, Yamagata, Japan; 4 United Graduate School of Agricultural Sciences, Iwate University, Morioka, Iwate, Japan; Bangabandhu Sheikh Mujibur Rahman Agricultural University, BANGLADESH

## Abstract

*Aucuba japonica* Thunb. is an evergreen understory shrub that grows naturally at a mine site. The mine soil contains high concentrations of heavy metals, and *A*. *japonica* appears to maintain detoxification mechanisms against heavy metals in the study site’s understory. This study aimed to investigate the heavy metal tolerance mechanisms in *A*. *japonica*, considering the possible roles of arbuscular mycorrhizal and root-endophytic fungi. We conducted fieldwork in summer (canopy-foliation season) and winter (canopy-defoliation season) to measure the heavy metal concentrations in leaves, branches, and roots and analyze possible detoxicants in the roots. The infection rates of arbuscular mycorrhizal and root-endophytic fungi were evaluated via microscopic observation, and heavy metal (Zn) localization in *A*. *japonica* roots was observed using confocal laser scanning microscopy. Field analysis showed that *A*. *japonica* accumulated excessive Zn and produced aucubin and citric acid in the roots in both summer and winter. Zn localization observations clarified that Zn was distributed in thickened epidermal and cortical cell walls, suggesting that the cell walls functioned as Zn deposition sites, reducing Zn toxicity. It was further clarified that Zn was contained within cortical cells, indicating that Zn might be detoxified by aucubin and citric acid. Arbuscular mycorrhizal and root-endophytic fungi within cortical cells adsorbed Zn on fungal cell walls, indicating that these fungi would reduce Zn content within root cells and might alleviate Zn toxicity. Our results indicated that *A*. *japonica* would maintain Zn tolerance in both summer and winter via Zn immobilization in the cell walls and production of aucubin and citric acid, and that arbuscular mycorrhizal and root-endophytic fungi might play important roles in the Zn tolerance of *A*. *japonica*.

## Introduction

Heavy metals, which refer to metals with densities of more than 5 g/cm^3^ [1], naturally occur in rocks and can be found around mine sites, agricultural lands, and urban industrial areas due to industrial activities [2]. Some heavy metals, such as Cu and Zn, are essential for plant growth, but excess heavy metals (both essential and non-essential) in plant tissues can cause a range of toxicity symptoms such as chlorosis, root-tip browning, and plant growth retardation [3–5]. These symptoms result from oxidative damage to biomolecules or inhibition of essential enzymatic reactions in plant metabolism [6, 7]. Although excessive amounts of heavy metals in soils can be toxic to plants, some plants grow well on heavy metal deposits without showing toxicity symptoms. These plants are thought to possess specific detoxification mechanisms for heavy metals acquired during their evolution [8].

Several detoxification mechanisms are involved in heavy metal tolerance in plants: (i) immobilization of metal ions in cell walls, (ii) impeded permeation across cell membranes, (iii) formation of chelates by ligands, (iv) metal compartmentalization in vacuoles, and (v) active export of metals into the apoplast [9]. Among these heavy-metal tolerance mechanisms, cell wall immobilization is one of the main strategies for reducing heavy metal toxicity in plants, and cell walls function as excretory sites for heavy metals in trees and herbaceous plants [10]. Additionally, detoxicant production is a vital detoxification mechanism in plant cells. Plants produce various types of primary and secondary metabolites with antioxidant activity or high affinity to metals. These detoxicants directly scavenge reactive oxygen species or prevent heavy metals from binding to biomolecules [11, 12]. Therefore, the detoxification mechanisms described above are considered necessary for plants to survive under heavy metal stress.

Under heavy metal polluted soil, soil microflora would be specifically formed compared with non-polluted soil and plant-microbe interaction would evolve symbiotically [13, 14]. Several studies have shown that microbes in the rhizosphere, such as arbuscular mycorrhizal fungi (AM fungi) and root-endophytic fungi (root endophytes; which, for all or part of life cycle, invade the tissues of living plants and cause an apparent and asymptomatic infections entirely in plant tissues [15]), are important for heavy metal tolerance in plants. Under heavy metal stress, AM fungi can reduce heavy metal uptake in shoots, resulting in the alleviation of heavy metal toxicity [16]. Field surveys suggest that AM fungi are associated with a majority of the plants in the Zn, Cu, Pb, and Ni contaminated soils and support the plants to survive under heavy metal stress [17]. On the other hand, root endophytes are known to confer a range of habitat-adapted stress tolerance to plants, including heavy metal, salt, drought, and pathogen tolerance [18]. Under heavy metal stress, root endophytes can improve heavy metal tolerance in hosts by restricting the translocation of heavy metals from roots to shoots, suggesting fungal immobilization of heavy metals within the root system [19]. Some endophytes may also immobilize heavy metals in their hyphae or soil to reduce their uptake by plant roots [20]. A recent study has shown that root endophytes can enhance plant growth by promoting nutrient uptake and reducing heavy metal concentrations in plant tissues [21]. In rhizosphere, some microbes alleviate metal stress by reducing metal uptake and strengthening antioxidant system [22]. These results indicate that symbiosis with microbes in the rhizosphere cannot be separated from heavy metal tolerance in plants. However, few studies have investigated heavy metal detoxification mechanisms in plants, considering the roles of microbes in the rhizosphere.

*Aucuba japonica* Thunb. is an evergreen understory shrub that naturally grows in the Hitachi Mine forest, once a major copper mine in Japan. The forest soil contains high concentrations of heavy metals (Cd, Cu, Pb, and Zn) and is acidic [21]. *A*. *japonica* is one of the dominant species found throughout the mine area. However, its heavy metal tolerance mechanisms have not yet been clarified. *A*. *japonica* is known to maintain photosynthetic activity throughout the year on the forest floor, where light conditions change with shedding and flushing of leaves in the canopy [23]. This may influence detoxicant production in *A*. *japonica* qualitatively and quantitatively. *A*. *japonica* produces high concentrations of aucubin, an iridoid glycoside with antioxidant activity [24, 25]. Therefore, aucubin might function as one of the primary heavy metal detoxicants. To evaluate the heavy metal tolerance of *A*. *japonica*, it is important to consider the seasonal variations in physiological activities, possible detoxicant production, and heavy metal accumulation.

The purpose of this study was to investigate the heavy metal tolerance mechanisms in *A*. *japonica*, considering the possible roles of AM fungi and root endophytes. We focused on summer (canopy-foliation season) and winter (canopy-defoliation season) to evaluate heavy metal tolerance in *A*. *japonica*. Fieldwork was conducted in July (summer) and January (winter) for 2 years. We analyzed the heavy metal concentrations in *A*. *japonica* (leaves, branches, and roots) and possible detoxicants (aucubin and organic acids) in *A*. *japonica* roots. In addition, the infection rates of AM fungi and root endophytes were evaluated using optical microscopy, and the heavy metal (Zn) localization and fungal structures in the root sections were observed by confocal laser scanning microscopy (CLSM). Finally, we discussed the possible detoxification mechanisms in *A*. *japonica*, considering the roles of AM fungi and root endophytes. By comparing the summer and winter results, we also discussed the sustainability of chemical defenses, such as aucubin and organic acid production, achieved through continuous photosynthesis in the study site’s understory.

## Materials and methods

### Study site

The study site was located in a mixed deciduous forest around the Hitachi Mine in Ibaraki Prefecture, Japan (36°37′ N, 140°38′ E). The mean annual temperature was 14.9°C, and the mean monthly temperature was highest in August (25.7°C) and lowest in January (4.6°C) during the study period (January 2016 to December 2018) [26]. The annual precipitation was 1,282 mm during the study period [26]. The experimental plot was established on each side of the forest road (north side, 600 m^2^; south side, 288 m^2^; total, 888 m^2^), and the number of trees over 1.3 m in height were as follows: *A*. *japonica*, 217; *Eurya japonica* Thunb., 27 (planted tree); *Clethra barbinervis* Sieb. et Zucc., 21; *Cornus macrophylla* Wall., 14; *Callicarpa japonica* Thunb., 8. The upper tree species were *E*. *japonica*, *C*. *barbinervis*, and *C*. *macrophylla*. The relative light intensity at 1.3 m above the ground in the experimental plots was evaluated in summer (July 2017) and winter (January 2018) using a light meter (model LI-250; Li-COR Inc., Lincoln, Nebraska, USA), and it was 0.4% (SE, ± 0.07) and 24.4% (SE, ± 4.4), respectively.

### Sampling of root-zone soil and plants, and measurements of photosynthetic rate

In October 2016, root-zone soil (150 mm × 150 mm × 5 mm, the soil including *A*. *japonica* roots) was collected from 10 arbitrarily selected *A*. *japonica* individuals (male, *n* = 5; female, *n* = 5; average tree height ± SE, 3.6 ± 0.2 m; average age ± SE, 27.7 ± 1.9 years old confirmed via counting the winter bud scars).

Plant samples (leaves, branches, and roots) were collected in July 2016 and January 2017 from 10 *A*. *japonica* individuals, as described above. The plant samples were used for the heavy metal concentration analysis, and some of the roots were used for AM fungi and root endophyte observation (the fieldwork schedule is shown in S1 Fig). After sampling in the first year, another 10 *A*. *japonica* individuals (male, *n* = 5; female, *n* = 5; average tree height ± SE, 3.1 ± 0.1 m) were arbitrarily selected since *A*. *japonica* trees do not have many roots and seem to be vulnerable. In July 2017 and January 2018, plant samples (leaves, branches, and roots) were collected from each individual and used for heavy metal concentration analysis. Some of the roots were used for detoxicant analysis and Zn localization observations (S1 Fig).

Photosynthetic light-response curves were measured in fully expanded young leaves of 10 field-growing individual trees in summer (July 2017) and winter (from December 2017 to February 2018) using a portable open gas exchange system (LI-6400; LI-COR Inc.) in a field setting. The measurements were conducted with 400 μmol mol^-1^ CO_2_ in the inlet gas stream, and leaf temperatures were not regulated (the detailed method is shown in S1 Protocol). Ten replicates were averaged, and their standard errors were calculated. The 10 individual trees were the same as those used for detoxicant measurements.

### Analysis of soil properties and heavy metal concentrations in *A*. *japonica*

Before analysis, the soil was air-dried for 2 weeks and passed through a 2-mm sieve. Heavy metal concentrations were determined using inductively coupled plasma optical emission spectrometry (ICP-OES; Optima 7300DV; PerkinElmer, Waltham, MA, USA) after digestion in concentrated HNO_3_ and HClO_4_ (1:4 v/v) at 130°C. Exchangeable Cd, Cu, Pb, and Zn were measured according to a previously described method [27]. Exchangeable Cd, Cu, and Zn were extracted from the soil (1.0 g) using 0.05 M Ca(NO_3_)_2_ (10 mL) by shaking at 150 rpm for 24 h at 30°C. Exchangeable Pb was extracted from the soil (1.0 g) using 1 M CH_3_COONH_4_ (pH 4.5, 10 mL) by shaking at 150 rpm for 1 h at 30°C. After filtration, heavy metal concentrations were quantified using ICP-OES. Soil pH (H_2_O) was measured using a pH meter (F-22; Horiba, Kyoto, Japan; soil: water, 1:2.5 w/v). The cation exchange capacity was measured using the semi-micro Schollenberger method [28]. Organic C and total N were measured using a C/N analyzer (UNIQUBE; Elementar, Langenselbold, Germany). Ten replicates for heavy metal concentrations, exchangeable heavy metals, and soil pH (H_2_O) were averaged, and their respective standard errors were calculated. Cation exchange capacity, organic C, and total N were obtained by a single analysis of the mixed soil from 10 replicates of root-zone soil.

The plant samples were carefully washed with running water and rinsed with deionized water thrice. The leaves were separated into current-year and 1-year-old leaves, according to a previous report [29]. After drying at 80°C for 48 h, the plants were ground using an agate mortar and digested in concentrated HNO_3_ at 130°C. After filtration, the solutions were analyzed using ICP-OES, and the heavy metal concentrations were determined. Ten replicates were averaged, and their standard errors were calculated. The heavy metal concentrations in leaves collected in July 2016 and January 2017 were averaged from six replicates (male, *n* = 3; female, *n* = 3) because in the first analysis, we needed to determine whether there were differences between the heavy metal concentrations in current-year and 1-year-old leaves. In the second year, we separated all leaves into current-year and 1-year-old leaves, considering the results obtained in the first year of research.

### Identification and quantification of possible detoxicants in *A*. *japonica* roots

For possible detoxicant determination in roots, the collected roots were thoroughly washed and extracted in methanol in the dark at 25°C for a week. The root extracts were dried in a centrifugal evaporator and dissolved in 1 mL of 50% methanol. For aucubin identification, liquid chromatography-mass spectrometry (LC/MS; LCMS-2020; Shimadzu, Kyoto, Japan) was used under the following conditions: column, Mightysil RP-18 (4.6 mm i.d.×150 mm); eluent, ultrapure water containing 0.1% formic acid (v/v); flow rate, 0.2 mL/min; column temperature, 40°C; detected wavelength, 210 nm. The MS conditions were as follows: interface, electrospray ionization (ESI) in positive ion mode; interface temperature, 350°C; desolvation line temperature, 200°C. The total ion chromatogram was recorded at mass/charge ratios (*m*/*z*) ranging from 50 to 500. The retention time and mass spectra were compared with an aucubin standard (FUJIFILM Wako Pure Chemical, Osaka, Japan).

Organic acids in the root extracts were purified using a weakly basic anion exchanger column, Toyopak-DEAE M column (1 mL; Tosoh Corporation, Tokyo, Japan), and dried according to a previously described method [30]. The purified samples were dissolved in 200 μL of pyridine and trimethylsilylated using 100 μL of *N*-methyl-*N*-(trimethylsilyl) trifluoroacetamide (MSTFA; Thermo Fisher Scientific, Millersburg, PA, USA) at 60°C for 15 min. The reactants were analyzed using gas chromatography-mass spectrometry (GC/MS; GCMS-QP2010 Ultra; Shimadzu, Kyoto, Japan), as described in [30]. The mass spectra of the main peaks were compared with the spectra shown in the 9th edition of the Wiley Registry of Mass Spectral Data library (Wiley, NJ, USA). The main peaks were identified according to the retention time and mass spectrum of a citric acid standard (FUJIFILM Wako Pure Chemical) trimethylsilylated with MSTFA using the same procedure described above.

Aucubin and citric acid in the root extracts were quantified using HPLC under the following conditions: column, Inertsustain C18 (4.6 mm i.d.×250 mm; GLSciences, Tokyo, Japan); eluent, 10 mM NH_4_H_2_PO_4_ (pH 2.6, H_3_PO_4_); flow rate, 1.0 mL/min. Standard curves for aucubin and citric acid were prepared by dissolving the standard in 50% methanol. The concentrations of 10 replicates were averaged and expressed as mg⁄g FW (± SE).

### Microscopic observation of AM fungi and root endophytes

The roots collected in July 2016 and January 2017 were thoroughly washed and stained with trypan blue after autoclaving with 10% KOH (w/v) for 30 min and rinsed with 5% HCl for 5 min. To determine the infection rates of AM fungi, fungal structures, such as *Paris* type, *Arum* type, vesicle, and hyphae, were observed using an optical microscope (100× and 400× magnification, CX21; Olympus, Tokyo, Japan) at 150 intersections per individual—infection rates were calculated according to the gridline-intersect method [31]. To determine the infection rates of root endophytes, fungal structures, such as microsclerotia and hyphae [32], were observed in the same procedure as described above. Ten replicates were averaged, and their standard errors were calculated.

### Observation of Zn localization in *A*. *japonica* roots

According to the *A*. *japonica* heavy metal concentrations results, the roots contained the highest concentration of Zn compared with the other metals in both seasons. Therefore, we focused on Zn. The roots collected in July 2017 and January 2018 were thoroughly washed as described above, fixed in 5% glutaraldehyde in 0.1 M sodium phosphate buffer (pH 7.2), and stored at 4°C. Before use, the fixed roots were thoroughly rinsed with deionized water, followed by 10 mM ethylenediaminetetraacetic acid disodium (Na_2_EDTA) for 5 min, and ultrapure water for 5 min. The roots were embedded in 4% agar, and 80 μm-thick transverse sections were prepared using a microtome (REM-710; Yamato Kohki Industrial Co., Miyazaki, Japan). To observe Zn localization, Zinpyr-1, which has high selectivity for zinc [33] and emits fluorescence at 520 nm with an excitation maximum at 490 nm, was applied as a Zn fluorescent staining reagent. The root sections were stained with 10 μM Zinpyr-1 (C_46_H_36_C_l2_N_6_O_5_; Santa Cruz Biotechnology, Dallas, TX, USA) in 0.1% dimethyl sulfoxide (DMSO) for 1 h in the dark and rinsed with ultrapure water for 5 min, according to a previously described method [34]. Zinc localization was observed by CLSM (FV10i, Olympus) using an excitation wavelength of 473 nm with an emission filter (Alexa Fluoro 488), and the green fluorescence emitted from Zn-Zinpyr-1 complex was observed. To confirm that the fluorescence was derived from the Zinpyr-1 complex with Zn, TPEN (N, N, N′, N′-tetrakis (2-pyridylmethyl) ethylenediamine) was applied according to a previously described method [35]. The root sections were placed in 1 mM TPEN with 0.1% DMSO for 2 h in the dark before staining with the Zinpyr-1 solution. The root sections were observed using CLSM under the same conditions described above, and fluorescence derived from Zinpyr-1 was not observed. For clarification of AM fungi and root endophyte distribution in the root sections, the fungal structures were stained using trypan blue and observed using optical microscopy. The treatment described above was conducted for both samples collected in summer and winter, and the Zn localization patterns were compared between the root sections prepared from summer and winter samples.

### Statistical analysis

Statistical analysis was conducted using the SPSS statistical software for Windows (ver. 24.0; SPSS Inc., Chicago, IL, USA). The differences between summer and winter for each variable were evaluated using the Wilcoxon signed-rank test after conducting Shapiro-Wilk test for evaluating normality. For the infection percentages of AM fungi and root endophytes, the differences between summer and winter were calculated in the same way as described above, after square roots of the percentages were transformed to their arcsine. Differences were considered significant at *P* < 0.05.

### Ethics statement

The study site was located in the Japanese National Forest. Our fieldwork activities, including observations and collections of plant materials and soil, were permitted by the Ibaraki District Forest Office. Any endangered or protected species were not involved.

## Results

### Photosynthetic rate in the field

The net photosynthetic rate was significantly higher in summer than in winter at 300 μmol m^-2^s^-1^ (*P* = 0.028), 200 μmol m^-2^s^-1^ (*P* = 0.007), 100 μmol m^-2^s^-1^ (*P* = 0.022), 50 μmol m^-2^s^-1^ (*P* = 0.017), and 30 μmol m^-2^s^-1^ (*P* = 0.047) (S2 Fig). Even at low air temperatures in winter, there were no significant differences in the net photosynthetic rates at 1500 μmol m^-2^s^-1^ (*P* = 0.721), 1000 μmol m^-2^s^-1^ (*P* = 0.414), 750 μmol m^-2^s^-1^ (*P* = 0.093), 500 μmol m^-2^s^-1^ (*P* = 0.203), 15 μmol m^-2^s^-1^ (*P* = 0.878), 8 μmol m^-2^s^-1^ (*P* = 0.074), and 0 μmol m^-2^s^-1^ (*P* = 0.878) between summer and winter in the field measurements (*n* = 10, S2 Fig).

### Heavy metal concentrations in root-zone soil and *A*. *japonica*

**Fig 1:**
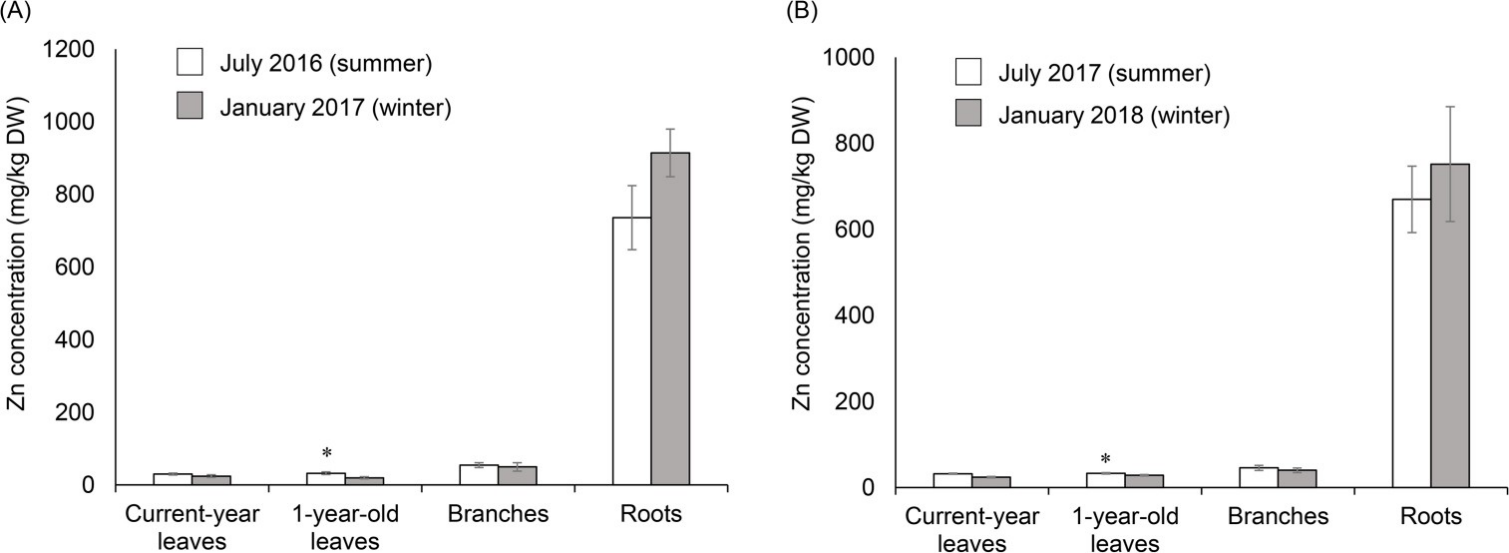
Zn concentrations in *A*. *japonica*. (A) Zn concentrations in *A*. *japonica* in July 2016 and January 2017, and (B) Zn concentrations in *A*. *japonica* in July 2017 and January 2018. Zn concentrations are expressed as means ± standard error (current-year and 1-year-old leaves in July 2016 and January 2017, *n* = 6; other parts, *n* = 10). Differences were evaluated by Wilcoxon signed-rank test: *, *P* < 0.05.

Root-zone soil contained high Cd, Cu, Mn, Pb, and Zn concentrations compared with the general forest soil [36], and the soil was acidic (S1 Table). The roots contained higher Cd, Cu, Pb, and Zn concentrations than the aboveground parts (current-year and 1-year-old leaves) and did not show seasonal variation, except for Mn (*P* = 0.022) (Fig 1 and S2 Table). Moreover, Zn concentrations in the roots were higher than those of the other heavy metals throughout the sampling period (Fig 1 and S2 Table). In the aboveground parts, Mn concentrations in the 1-year-old leaves were higher than those of the other metals throughout the sampling period (S2 Table). The leaves also contained Cu, and Zn throughout the sampling period and the Pb and Cd concentrations were below quantification limit (Fig 1 and S2 Table). The branches also contained Cu, Mn, and Zn throughout the sampling period and Pb was detected only in January 2017, and the Cd concentrations were below detection limit (Fig 1 and S2 Table). Zn showed seasonal variations; the concentrations in 1-year-old leaves were significantly higher in July 2016 (summer) than in January 2017 (winter) (*P* = 0.028), and higher in July 2017 (summer) than in January 2018 (winter) (*P* = 0.047) (Fig 1). Mn also showed seasonal variations; the concentration in current-year leaves was significantly higher in January 2017 (winter) than in July 2016 (summer) (*P* = 0.046) and higher in January 2018 (winter) than in July 2017 (*P* = 0.022), and the concentration in 1-year-old leaves was significantly higher in July 2017 than in January 2018 (*P* = 0.007) (S2 Table). Copper exhibited seasonal variations; the concentrations in current-year leaves were higher in January 2017 (winter) than in July 2016 (summer) (*P* = 0.028), and the concentration in branches was higher in July 2017 (summer) than in January 2018 (winter) (*P* = 0.022).

### Possible detoxicants in *A*. *japonica* roots

LC/MS analysis detected aucubin in *A*. *japonica* roots. Aucubin concentrations were higher than that of citric acid and showed seasonal variation; the concentration in summer (July 2017; 22.9 ± 1.1 mg/g FW) was significantly higher than in winter (January 2018; 19.8 ± 1.6 mg/g FW; *P* = 0.037; Wilcoxon signed-rank test; *n* = 10). GC/MS analysis detected citric acid in *A*. *japonica* roots. Citric acid concentration was significantly higher in winter (January 2018; 1.3 ± 0.1 mg/g FW) than in summer (July 2017; 0.8 ± 0.1 mg/g FW; *P* = 0.017; Wilcoxon signed-rank test; *n* = 10).

### AM fungi and root endophyte infection rates

Microscopic observation revealed that the roots were colonized by AM fungi and root endophytes, and the infection rates of AM fungi (*Paris* type, *Arum* type, vesicle, and hyphae) did not show seasonal variation (*P* = 0.575, 0.059, 0.173 and 0.646, respectively, Wilcoxon signed-rank test, n = 10, Table 1). AM fungi primarily produced hyphal coils, classified as *Paris* type; however, arbuscules and vesicles were not frequently observed. Root endophytes produced microsclerotia, and the infection rates of root endophytes (microsclerotia and hyphae) did not show seasonal variation (*P* = 0.646 and 0.333, respectively, Wilcoxon signed-rank test, n = 10, Table 1).

**Table 1:** AM fungi and root endophyte infection rates.

Month	AM fungi (%)												Root endophytes (%)					
	*Paris* type			*Arum* type			Vesicle			Hyphae			Microsclerotia			Hyphae		
July 2016 (summer)	63.9	±	7.6	0.8	±	0.5	16.0	±	3.2	58.2	±	9.3	19.7	±	5.3	46.8	±	10.3
January 2017 (winter)	68.3	±	5.6	0.1	±	0.1	11.6	±	2.2	64.5	±	7.4	20.3	±	3.9	56.1	±	8.9

The percentages are shown as means ± standard error (*n* = 10).

### Zn localization in *A*. *japonica* roots

**Fig 2:**
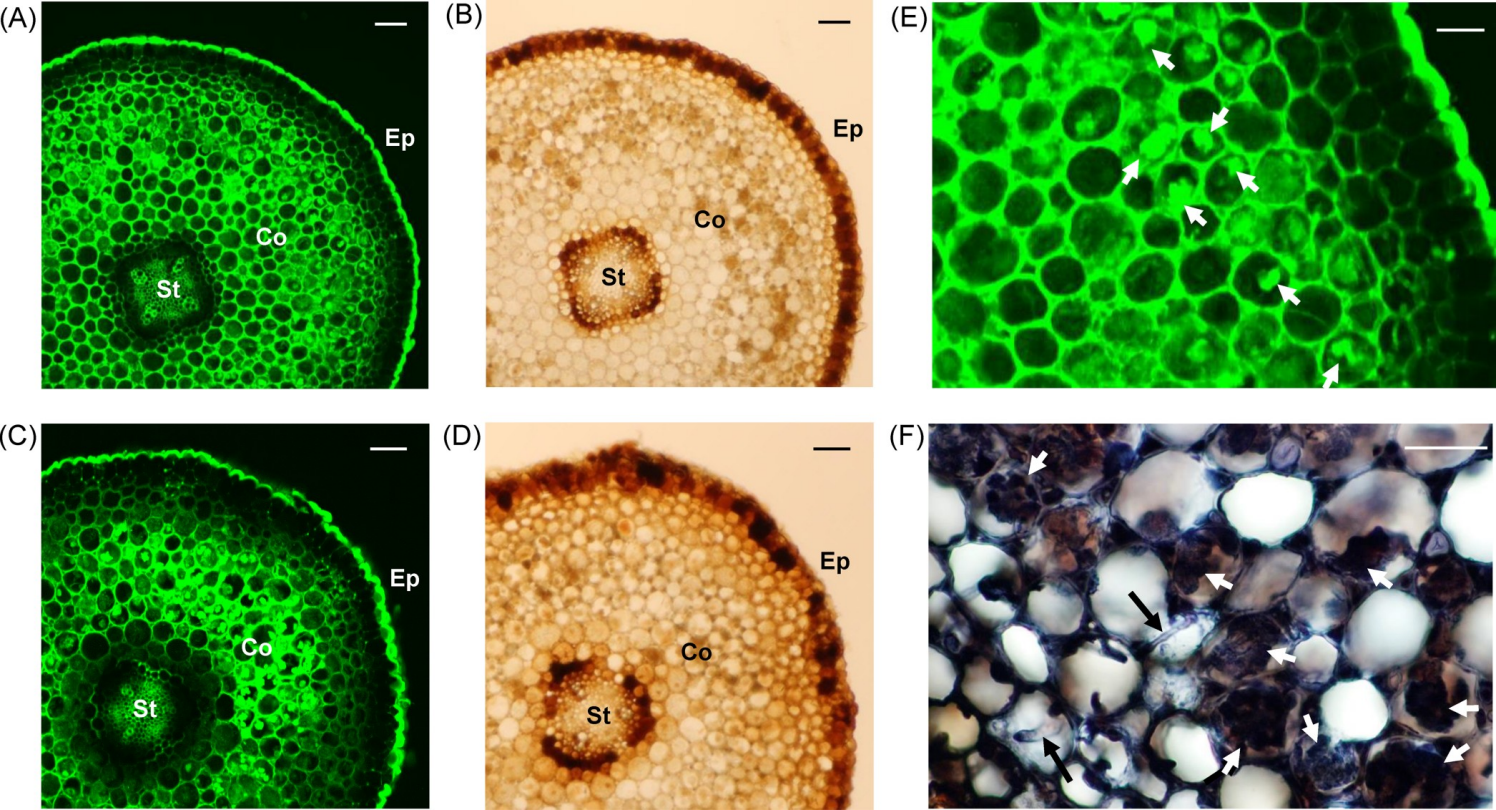
Zn localization in the roots of *A*. *japonica*. (A) Zn localization in roots collected in July 2017 (summer). (B) Optical microscopically observed root sections collected in July 2017 (summer). (C) Zn localization in roots collected in January 2018 (winter). (D) Optical microscopically observed root sections collected in January 2018 (winter). (E) The extended images of the root cortex and epidermis of Fig 2A. (F) Trypan-blue stained fungal structures in the roots collected in July 2017. The green color indicates fluorescence derived from the Zinpyr-1 complex with Zn. St, stele; Co, cortex; Ep, epidermis. White arrows indicate the fungal structures, and black arrows indicate fungal hyphae. The Scale bars in Fig 2A–2D represent 100 μm, and Fig 2E and 2F represent 50 μm.

Zinpyr-1 complex fluorescence was mainly observed in the epidermal and cortical cell walls of roots (Fig 2A and 2C). The epidermal cell walls were thicker than the other cell walls and emitted intense fluorescence (Fig 2A, 2C and 2E). In the cortex, weak fluorescence was observed in the intercellular spaces (Fig 2A, 2C and 2E). Additionally, trypan blue staining showed fungal structures colonizing cortical cells (Fig 2F), and Zinpyr-1 complex fluorescence was observed in the fungal structures (Fig 2E). However, it was difficult to distinguish AM fungi from root endophytes accurately because the fungal mycelia were extremely overlapped in the root sections under CLSM. Weak fluorescence was also observed within cortical cells (Fig 2A, 2C and 2E). The localization pattern of fluorescence in the root sections was mostly consistent between summer and winter (Fig 2A and 2C).

## Discussion

Although the root-zone soil contained high heavy metal concentrations, *A*. *japonica* did not transfer heavy metals, except Mn, to the leaves and accumulated Zn, Pb, Cu, and Cd in the roots throughout the study period. The Mn concentrations in 1-year-old leaves were higher than those of the other metals, but the Mn concentrations did not exceed the critical toxic levels (200–3500 mg/kg DW [37]). Mn is known as essential element, functioning as metalloenzymes, such as in photosystem Ⅱ, and Mn translocation to leaves has been observed in other tree species, growing on normal forest soil [38]. Therefore, Mn translocation to leaves appeared to be part of nutrient acquisition for physiological activities, such as photosynthesis in *A*. *japonica*. In the roots, Zn concentrations were higher than those of the other metals contained in the roots and exceeded the critical toxic level in plants (Zn, 100–500 mg/kg DW [2]). Zn is an essential element for plant growth, but excess Zn causes oxidative damage to biomolecules, resulting in the inhibition of root elongation [39]. However, *A*. *japonica* grew well at the research site without any Zn toxicity symptoms. Therefore, *A*. *japonica* should have detoxification mechanisms for excessive amounts of Zn in the roots. By observing Zn localization, it was confirmed that Zn accumulated in the thickened epidermal and cortical cell walls of *A*. *japonica* roots (Fig 2A, 2C and 2E), indicating that the cell walls function as Zn deposition sites in the roots. Cell walls contain pectin, a family of polysaccharides formed by galacturonic acids [40], which can adsorb divalent and trivalent cations, including Zn [41]. Few studies have focused on Zn tolerance and cell wall functions in wild tree species, but it has been shown that *Populus tremula* sapling, grown in soil with added Cd, Cu, Pb, and Zn, exhibited metal tolerance by accumulating large amounts of Cu and Zn in the fine root cell walls [42]. Our results suggest that the cell walls in *A*. *japonica* roots function as Zn deposition sites and alleviate Zn toxicity by preventing Zn entry into the cytosol. In addition, cell-wall thickening is known to be a defensive response that increases heavy metal immobilization capacity [43]. Cell wall thickening has been observed in several plant species, such as *Triticum aestivum*, grown under excessive Zn levels [44], and *Populus tremula*, grown under excessive Pb levels [43]. It has been shown that thickened cell walls in the roots of *Vicia faba* growing in mine tailing soil prevented excessive Pb and Zn entry within root cells, resulting in metal toxicity alleviation [45]. Therefore, it is possible that cell-wall thickening in the epidermal cells of *A*. *japonica* roots might be induced by heavy metal stress at the study site and contribute to the reduction of excessive Zn entry within roots.

Metabolite analysis showed that *A*. *japonica* roots produced possible detoxicants, such as aucubin and citric acid. It has been demonstrated that aucubin in aqueous solution scavenges reactive oxygen species, O_2_^-^ and ·OH even at low concentrations [25, 46]. Iridoid glycosides, including aucubin, are considered to have roles during oxidative stress under abiotic stress condition [47]; for example, under drought stress, aucubin and other iridoid glycoside’s concentrations significantly increased with the elevation of free radical levels in *Scrophularia ningpoensis* [48]. Under exposure to Ni- and Al-oxides nanoparticles, iridoid glycosides, phenolics, and flavonoids contents were significantly increased with the accumulation of reactive oxygen species in *Nigella arvensis*, suggesting the possible roles of iridoid glycosides during metal stress [49]. In our study, the aucubin concentrations (summer, 132 mg/g DW; winter, 114 mg/g DW; calculated using root water content) in *A*. *japonica* roots were considerably higher than those of general antioxidants in plants; for example, the concentrations of antioxidative total phenolics in tree leaves were approximately 20 to 40 mg/g DW [50], and aucubin concentration in *Plantago* species and *Eucommia ulmoides* (abundant in aucubin) were approximately 7 to 20 mg/kg DW [51, 52]. This suggests that *A*. *japonica* constantly produces a high concentration of aucubin in roots, which can scavenge reactive oxygen species in plants. In order to confirm that aucubin functions as antioxidant for heavy metals in *A*. *japonica* roots, we need to monitor reactive oxygen species and lipid peroxidation products in *A*. *japonica* roots in the field. In summary, aucubin might function as a vital detoxicant for heavy metal tolerance in *A*. *japonica* roots due to its abundance and constant production in both summer and winter. This might be attributed to the sustainability of photosynthesis in *A*. *japonica*; *A*. *japonica* maintains photosynthetic activity in summer and winter because it acclimates the leaf’s anatomical structures to acquire sunlight under deciduous trees [53]. At our study site, *A*. *japonica* maintained photosynthetic activity in summer and winter (S2 Fig). This is consistent with the results of previous study [23], suggesting that *A*. *japonica* might acquire sufficient carbon, which can be supplied to the roots in summer and winter, resulting in constant aucubin production.

Although the citric acid concentrations were lower than that of aucubin, citric acid is known as a vital detoxicant for many plants because it can form stable complexes with various metal ions, such as Al, Cd, Cu, Fe, Pb, and Zn [54]. Many researches have suggested that citric acid function as vital detoxicant for metals inside or outside the roots of plants; for example, previous researches pointed out the positive relation between organic acid contents and metal tolerance in plants growing on metalliferous soils [55–58], and advanced technique using extended x-ray absorption spectroscopy suggested that Zn was accumulated in the roots of Zn hyperaccumulator, *Arabidopsis halleri*, binding to oxygen atoms possibly originated from malic and citric acid [59]. Comparing the citric acid contents of plant roots in various nutrient status (mean value, 0.78 mg/kg FW [60]), the citric acid contents in *A*. *japonica* roots were higher than the mean value. Although we did not confirm the direct contribution of citric acid to metal stress in *A*. *japonica*, citric acid may function as one of the detoxicants for Zn or other metals within *A*. *japonica* roots.

Under heavy metal stress conditions, microbial interactions in the rhizosphere play important roles in heavy metal tolerance of plants. In *A*. *japonica* roots, fungal structures within cortical cells adsorbed Zn (Fig 2E and 2F), suggesting that AM fungi and root endophytes would function as Zn deposition sites. The cell walls of fungal species are rich in chitin, chitosan, and melanin, which can adsorb a wide range of heavy metals [61]. Previous researches have shown that accumulation of metals on or within mycorrhizal fungi serve as a protective function for roots of forest trees; for example, ectomycorrhizal fungal hyphae have been shown to prevent Zn entry into *Betula* spp. roots by adsorbing Zn on extraradical hyphal cell walls and to alleviate Zn toxicity [62]. The ectomycorrhizal mantle hyphae and hartig net accumulated high concentrations of Zn, suggesting that fungal tissues were the main accumulation sites for Zn in *Picea abies* and these fungal tissues decreased Zn transfer from the fungus to the root [63]. Although our research does not directly confirm the infection effect of these fungi to the metal tolerance in *A*. *japonica*, our results suggest that these fungi might function as Zn deposition site and involve in metal tolerance of *A*. *japonica*. In order to confirm the effects of root endophytes and AM fungi on metal tolerance in *A*. *japonica*, we need to conduct inoculation tests of *A*. *japonica* with root endophytes and AM fungi isolates. However, we could not use the seedlings for the inoculation test, because the seedlings would have different physiological characteristics from mature *A*. *japonica*; for example, *A*. *japonica* seedlings did not highly accumulate heavy metals compared with mature trees (S3 Table), indicating different heavy-metal tolerance mechanism from mature trees. We need to take into account the inoculation test using the cuttings for example, after the confirmation of metal accumulation patterns of the cuttings grown in the mine soil, although we cannot eliminate all microbes by the sterilization. We will have several suggestions to the Zn tolerance related with root endophytes and AM fungi in the mature trees via the inoculation test.

## Conclusion

We found that *A*. *japonica*, naturally growing in the mine soil, accumulated Zn up to toxic levels in roots and maintained Zn tolerance in summer and winter via Zn immobilization in the cell walls and production of possible detoxicants (aucubin and citric acid). Additionally, AM fungi and root endophytes adsorbed Zn in fungal cell walls, which might reduce Zn toxicity within the roots and restrict the Zn translocation to the shoots. *A*. *japonica* is an evergreen understory shrub with high shade tolerance, growing in middle- to late-successional forests. Under heavy metal stress, it might be better to consider the trade-off theory between growth and defense [64]. According to field research using forty-seven lowland rain forest tree species, shade-tolerant tree species could allocate high amounts of carbon resources to defense in mature leaves, especially chemical defense (condensed tannin production) compared with growth [65]. In addition, under the controlled shaded condition, a tropical forest canopy species, *Eusideroxylon zwageri* seedlings, invested carbon into defensive compounds such as condensed tannin and lignin compared with growth [66]. In fact, *A*. *japonica* might survive heavy metal stress by investing carbons in possible detoxicant production (aucubin) due to its slow growth (S3 Fig). This might be one of the reasons why *A*. *japonica* was established at our research site with high frequency. Our research is the first report on heavy metal tolerance in the sub-climax tree, *A*. *japonica*, although there have been several reports of heavy metal tolerance of pioneer tree species such as *Salix* spp. [67] and *Betula* spp. [68]. Therefore, we hope that research on the heavy-metal tolerance of sub-climax trees will be conducted because these trees would could be essential for sustainable greening at mine sites.

## Supporting information

S1 Protocol Measurement of photosynthetic light-response curves. (PDF)

S1 Data set (PDF)

S1 Fig Plant sampling month for each analysis. (PDF)

S2 Fig Photosynthetic light-response curve of *A*. *japonica* in summer and winter. The net photosynthetic rates are shown as the mean ± standard error (*n* = 10).
(PDF)

S3 Fig Growth rate of annual branch in *A*. *japonica*. Three branches were arbitrarily selected from each *A*. *japonica* (*n* = 10), and the growth rates (mm) of the annual branches were measured every month from July 2016 to January 2017 and from March 2017 to May 2017. The growth rates were expressed as means ± standard error (*n* = 10).
(PDF)

S1 Table Properties of the root-zone soil. Concentrations of heavy metals, exchangeable heavy metals, and pH (H_2_O) are shown as the mean ± standard error (*n* = 10). ND indicates that the concentration was below the detection limit.
(PDF)

S2 Table Cd, Cu, Mn, Pb, and Zn concentrations in *A*. *japonica*. Cd, Cu, Mn, Pb, and Zn concentrations are shown as means ± standard error (current-year and 1-year-old leaves in July 2016 and January 2017, *n* = 6; the other parts, *n* = 10). Differences were evaluated by Wilcoxon signed-rank test: *, *P* < 0.05; **, *P* < 0.01. ND indicates that the concentration was below the detection limit.
(PDF)

S3 Table Cd, Cu, Mn, Pb, and Zn concentrations in current-year *A*. *japonica* seedlings. Cd, Cu, Mn, Pb, and Zn concentrations are shown as means ± standard error (collected in April 2019, *n* = 5). *A*. *japonica* seedlings germinated from October to November in 2018. ND indicates that the concentration was below the detection limit.
(PDF)
