# Supplementary material for: Zn tolerance in the evergreen shrub, Aucuba japonica, naturally growing at a mine site: Cell wall immobilization, aucubin production, and Zn adsorption on fungal mycelia
Source: PLoS One. 2021 Sep 30;16(9):e0257690. doi: 10.1371/journal.pone.0257690 (PMC8483361; doi:10.1371/journal.pone.0257690)
Supplement: S1 Protocol — (PDF) [file pone.0257690.s001.pdf]

The H_2O and CO_2 concentrations in the gas stream within the system were determined using infrared absorption. The exposed photon flux density (PFD) was gradually decreased from 1500, 1000, 750, 500, 300, 200, 100, 50, 30, 15, 8, and 0 μmol $m^{-2}s^{-1}$ using red-blue light-emitting diodes.

Photosynthetic light-response curves were simulated using the following equation:

$$A_n = \frac{\Phi\, I + A_{max} - \sqrt{(\Phi\, I + A_{max})^2 - 4\,\theta\,\Phi\, I\, A_{max}}}{2\,\theta} - R_d$$

where A_n is the net assimilation rate, A_{max} is the maximum gross assimilation rate, I is the photon flux density (PFD), Φ is the initial slope, θ is the curvature, and R_d is the dark respiration rate. When $\theta = 0$, the fitting curve is converted into rectangular hyperbola equations; when $\theta = 1$, it is converted into Blackman equations. The values of Φ were 0.064 and 0.059 in summer and winter, respectively, and those of θ were 0.740 and 0.317 in summer and winter, respectively.
