# Supplementary material for: Zn tolerance in the evergreen shrub, Aucuba japonica, naturally growing at a mine site: Cell wall immobilization, aucubin production, and Zn adsorption on fungal mycelia
Source: PLoS One. 2021 Sep 30;16(9):e0257690. doi: 10.1371/journal.pone.0257690 (PMC8483361; doi:10.1371/journal.pone.0257690)
Supplement: S1 Data set — (PDF) [file pone.0257690.s002.pdf]

(A) Data set for aucubin and citric acid concentrations in *A. japonica* roots.

July 2017

Detoxicant (mg/g FW)	1	2	3	4	5	6	7	8	9	10
Aucubin	19.2	30.2	19.8	23.2	24.8	21.7	26.4	22.0	22.7	18.4
Citric acid	1.2	0.9	0.9	0.3	0.7	0.4	0.9	0.7	0.6	1.2

January 2018

Detoxicant (mg/g FW)	1	2	3	4	5	6	7	8	9	10
Aucubin	14.3	28.9	19.9	18.8	16.1	17.8	23.3	18.2	27.2	13.8
Citric acid	1.1	0.9	1.0	1.4	1.6	1.4	1.8	1.2	1.5	1.2

The numbers from 1 to 10 indicate the replications of *A. japonica*.

(B) Data set for Table 1: AM fungi and root endophytes infection rates.

July 2016

Replications	AM fungi (%)				Root endophytic fungi (%)	
	Paris type	*Arum* type	Vesicle	Hyphae	Microsclerotia	Hyphae
1	54.7	1.3	17.3	56.0	8.0	34.0
2	74.0	0.0	24.7	78.0	6.0	16.7
3	72.7	0.0	2.0	50.7	22.7	82.7
4	47.3	0.7	6.0	46.0	35.3	48.7
5	81.3	0.0	16.0	66.7	23.3	39.3
6	40.0	0.0	33.3	46.7	40.0	38.0
7	64.7	0.7	25.3	42.7	19.3	59.3
8	45.3	0.7	16.0	26.7	17.3	23.3
9	85.3	4.7	15.3	88.7	8.7	47.3
10	73.3	0.0	4.0	80.0	16.0	78.7

January 2107

Replications	Root endophytic fungi (%)				Root endophytic fungi (%)	
	Paris type	*Arum* type	Vesicle	Hyphae	Microsclerotia	Hyphae
1	56.0	1.3	10.0	49.3	10.7	48.7
2	71.3	0.0	22.7	66.7	18.0	43.3
3	59.3	0.0	4.7	53.3	28.7	81.3
4	72.0	0.0	22.7	77.3	16.0	30.0
5	68.0	0.0	16.0	76.7	28.0	66.0
6	71.3	0.0	8.0	74.0	33.3	79.3
7	69.3	0.0	7.3	66.7	25.3	58.7
8	81.3	0.0	13.3	90.7	19.3	60.7
9	87.3	0.0	9.3	45.3	15.3	66.0
10	46.7	0.0	2.0	45.3	8.7	26.7

The numbers from 1 to 10 indicate the replications of *A. japonica*.

(C) Data set for S1 Table: Properties of root-zone soil.

Properties	1	2	3	4	5	6	7	8	9	10
Total Cd (mg/kg)	3.0	3.1	3.6	3.3	3.4	2.5	3.4	3.3	3.3	2.8
Total Cu (mg/kg)	366.6	368.0	284.3	323.9	364.6	284.2	385.5	368.3	317.5	393.2
Total Mn (mg/kg)	586.1	952.2	2253.6	826.3	648.3	604.8	1015.2	875.5	724.3	547.3
Total Pb (mg/kg)	382.2	266.0	252.2	320.8	276.1	186.0	381.8	264.1	261.5	302.9
Total Zn (mg/kg)	100.9	107.8	131.8	140.4	116.3	93.2	215.6	116.7	118.8	88.0
Exchangeable Cd (mg/kg)	ND	ND	ND	ND	ND	ND	ND	ND	ND	ND
Exchangeable Cu (mg/kg)	4.0	5.9	1.0	1.2	2.1	2.3	2.1	2.3	2.3	5.0
Exchangeable Pb (mg/kg)	151.9	95.9	90.4	107.9	87.1	75.9	114.1	81.6	83.2	111.6
Exchangeable Zn (mg/kg)	18.2	5.5	16.7	16.2	10.7	10.4	14.8	5.3	20.7	8.6
pH (H_2O)	4.5	4.9	5.0	5.0	4.8	4.6	4.8	4.8	4.4	4.3

ND indicates that the concentration was below detection limit. The numbers from 1 to 10 indicate the replications of root-zone soil.

(D) Data set for S2 Table: Cd, Cu, Mn, Pb, and Zn concentrations in *A. japonica* collected in July 2016.

Current-year leaves

Element (mg/kg DW)	3	4	5	8	9	10
Cd	ND	ND	ND	ND	ND	ND
Cu	9.4	12.8	10.4	10.3	7.4	9.4
Mn	478.4	344.7	837.2	352.9	755.9	556.8
Pb	ND	ND	ND	ND	ND	ND
Zn	41.4	28.7	30.9	20.7	29.2	30.3

1-year-old leaves

Element (mg/kg DW)	3	4	5	8	9	10
Cd	ND	ND	ND	ND	ND	ND
Cu	4.7	7.8	7.5	17.9	6.3	5.0
Mn	1481.8	934.0	1385.0	544.7	2123.7	1447.8
Pb	ND	ND	ND	ND	ND	ND
Zn	35.1	28.8	34.2	22.6	25.7	47.1

Branches

Element (mg/kg DW)	1	2	3	4	5	6	7	8	9	10
Cd	ND	ND	ND	ND	ND	ND	ND	ND	ND	ND
Cu	220.9	19.2	16.1	54.1	19.1	30.9	28.6	21.9	76.7	25.1
Mn	449.3	337.2	262.2	176.0	338.8	407.3	381.6	224.2	486.7	437.1
Pb	ND	ND	ND	ND	ND	ND	ND	ND	ND	ND
Zn	72.8	57.0	52.9	27.2	30.1	51.3	40.3	48.7	64.8	98.8

Roots

Element (mg/kg DW)	1	2	3	4	5	6	7	8	9	10
Cd	36.1	23.2	6.8	33.3	51.9	24.1	27.5	21.1	42.4	29.3
Cu	53.8	53.5	59.9	62.6	91.1	56.9	104.1	113.2	78.1	69.7
Mn	251.0	240.1	162.7	206.1	182.1	124.7	255.7	167.1	322.9	133.9
Pb	228.6	202.4	16.5	129.2	500.0	190.5	341.2	127.7	182.2	284.8
Zn	722.3	577.2	453.6	1230.2	1101.9	586.9	630.5	799.3	908.3	349.2

ND indicates that the concentration was below detection limit. The numbers from 1 to 10 indicate the replications of *A. japonica*.

(E) Data set for S2 Table: Cd, Cu, Mn, Pb, and Zn concentrations in *A. japonica* collected in January 2017.

Current-year leaves

Element (mg/kg DW)	3	4	5	8	9	10
Cd	ND	ND	ND	ND	ND	ND
Cu	9.7	16.1	15.8	39.4	18.2	11.6
Mn	870.3	439.8	829.9	408.8	1616.8	873.3
Pb	ND	ND	ND	ND	ND	ND
Zn	21.8	20.0	14.3	38.1	21.9	30.1

1-year-old leaves

Element (mg/kg DW)	3	4	5	8	9	10
Cd	ND	ND	ND	ND	ND	ND
Cu	4.6	15.2	24.1	16.4	6.0	11.4
Mn	1355.2	635.3	1386.4	651.0	2827.8	1214.1
Pb	ND	ND	ND	ND	ND	ND
Zn	33.7	11.6	13.6	21.1	16.7	22.0

Branches

Element (mg/kg DW)	1	2	3	4	5	6	7	8	9	10
Cd	ND	ND	ND	ND	ND	ND	ND	ND	ND	ND
Cu	17.4	9.6	29.2	18.9	15.9	14.5	23.1	14.6	15.3	36.0
Mn	536.7	413.2	436.1	301.0	449.8	269.6	394.1	282.7	441.4	454.1
Pb	4.5	6.6	2.4	4.6	2.7	3.0	2.6	2.5	9.3	3.0
Zn	38.4	50.4	113.0	22.8	44.0	9.1	42.7	116.7	45.2	16.0

Roots

Element (mg/kg DW)	1	2	3	4	5	6	7	8	9	10
Cd	34.8	31.8	35.2	51.3	37.9	52.8	42.6	24.8	33.3	40.1
Cu	93.9	57.5	36.6	82.5	69.0	84.3	60.0	159.7	99.1	94.9
Mn	342.7	357.6	209.1	133.2	382.8	266.6	343.4	163.7	352.6	375.8
Pb	143.9	320.7	32.7	425.4	282.8	495.3	465.4	202.9	167.3	843.6
Zn	682.7	716.4	1074.1	1319.4	1114.2	972.5	897.9	680.5	849.6	836.7

ND indicates that the concentration was below detection limit. The numbers from 1 to 10 indicate the replications of *A. japonica*.

(F) Data set for S2 Table: Cd, Cu, Mn, Pb, and Zn concentrations in *A. japonica* collected in July 2017.

Current-year leaves

Element (mg/kg DW)	1	2	3	4	5	6	7	8	9	10
Cd	ND	ND	ND	ND	ND	ND	ND	ND	ND	ND
Cu	6.8	10.8	12.9	10.1	7.4	10.2	8.0	7.7	8.5	7.6
Mn	512.0	375.9	376.7	483.7	291.6	452.4	338.7	711.0	549.5	509.5
Pb	ND	ND	ND	ND	ND	ND	ND	ND	ND	ND
Zn	32.8	30.6	32.0	37.0	23.7	40.3	33.3	34.0	29.1	28.4

1-year-old leaves

Element (mg/kg DW)	1	2	3	4	5	6	7	8	9	10
Cd	ND	ND	ND	ND	ND	ND	ND	ND	ND	ND
Cu	6.0	7.0	8.8	8.1	9.3	8.1	6.2	8.5	6.5	8.6
Mn	1219.0	1416.8	985.9	1584.6	1268.2	883.5	992.8	2038.8	898.4	2133.3
Pb	ND	ND	ND	ND	ND	ND	ND	ND	ND	ND
Zn	28.7	24.9	30.1	39.0	30.3	27.1	37.1	35.1	35.1	40.3

Branches

Element (mg/kg DW)	1	2	3	4	5	6	7	8	9	10
Cd	ND	ND	ND	ND	ND	ND	ND	ND	ND	ND
Cu	42.0	19.8	56.7	33.3	26.6	33.6	47.8	14.6	17.5	58.4
Mn	211.3	322.1	200.9	471.4	242.7	316.8	322.0	226.1	324.1	223.8
Pb	ND	ND	ND	ND	ND	ND	ND	ND	ND	ND
Zn	44.2	29.3	46.4	90.9	44.4	41.5	56.4	22.2	44.3	37.4

Roots

Element (mg/kg DW)	1	2	3	4	5	6	7	8	9	10
Cd	27.0	25.1	24.2	38.2	14.3	11.5	20.4	55.8	16.4	22.3
Cu	52.7	99.3	53.7	165.4	96.3	153.6	86.8	151.9	81.3	65.1
Mn	250.1	185.9	196.0	87.4	72.4	68.4	400.7	266.9	237.8	97.1
Pb	312.7	221.0	147.9	863.0	48.2	112.5	221.8	186.4	27.0	311.6
Zn	931.1	796.5	701.6	738.2	338.9	364.1	835.4	1015.9	613.9	363.8

ND indicates that the concentration was below detection limit. The numbers from 1 to 10 indicate the replications of *A. japonica*.

(G) Data set for S2 Table: Cd, Cu, Mn, Pb, and Zn concentrations in *A. japonica* collected in January 2018.

Current-year leaves

Element (mg/kg DW)	1	2	3	4	5	6	7	8	9	10
Cd	ND	ND	ND	ND	ND	ND	ND	ND	ND	ND
Cu	8.2	6.5	8.1	6.3	7.9	8.2	16.4	15.0	6.6	10.5
Mn	734.4	534.2	686.4	497.2	742.0	514.1	650.3	778.6	383.1	791.7
Pb	ND	ND	ND	ND	ND	ND	ND	ND	ND	ND
Zn	29.0	19.3	21.8	21.3	36.1	23.7	26.1	25.0	17.6	20.1

1-year-old leaves

Element (mg/kg DW)	1	2	3	4	5	6	7	8	9	10
Cd	ND	ND	ND	ND	ND	ND	ND	ND	ND	ND
Cu	6.8	7.2	14.3	6.7	7.4	9.4	7.2	6.7	8.4	13.7
Mn	1225.3	767.2	927.0	791.6	1170.3	685.7	870.8	1191.4	690.0	1581.2
Pb	ND	ND	ND	ND	ND	ND	ND	ND	ND	ND
Zn	30.8	18.8	21.1	37.1	29.6	31.3	28.8	31.1	26.6	28.5

Branches

Element (mg/kg DW)	1	2	3	4	5	6	7	8	9	10
Cd	ND	ND	ND	ND	ND	ND	ND	ND	ND	ND
Cu	18.2	16.0	15.8	17.6	23.5	14.0	29.9	19.6	19.4	17.5
Mn	237.7	168.6	235.6	251.2	299.8	216.8	210.6	243.0	184.4	398.1
Pb	ND	ND	ND	ND	ND	ND	ND	ND	ND	ND
Zn	30.6	21.2	17.2	50.6	21.9	51.5	34.9	66.5	48.1	58.2

Roots

Element (mg/kg DW)	1	2	3	4	5	6	7	8	9	10
Cd	25.8	13.8	16.0	60.0	20.6	19.9	22.8	48.1	38.8	14.2
Cu	69.2	49.0	51.0	134.2	66.3	120.4	125.0	109.7	78.8	82.1
Mn	140.1	96.0	117.9	182.4	162.0	93.2	186.0	141.7	232.8	106.8
Pb	126.1	91.4	50.1	410.8	160.6	146.9	315.4	162.4	182.8	54.6
Zn	752.9	449.9	621.5	1735.7	384.4	561.4	733.2	755.0	1185.1	340.0

ND indicates that the concentration was below detection limit. The numbers from 1 to 10 indicate the replications of *A. japonica*.

(H) Data set for S3 Table: Cd, Cu, Mn, Pb, and Zn concentrations in current-year *A. japonica* seedlings.

Leaves

Element (mg/kg DW)	1	2	3	4	5
Cd	ND	ND	ND	ND	ND
Cu	11.5	13.2	12.9	17.1	13.9
Mn	124.2	70.6	1038.5	75.5	122.8
Pb	ND	ND	ND	ND	ND
Zn	37.4	55.1	71.3	34.5	31.6

Cotyledons

Element (mg/kg DW)	1	2	3	4	5
Cd	ND	ND	ND	ND	ND
Cu	ND	ND	ND	ND	ND
Mn	525.1	180.1	1954.8	190.6	358.0
Pb	ND	ND	ND	ND	ND
Zn	38.5	43.4	36.7	28.6	27.1

Hypocotyls

Element (mg/kg DW)	1	2	3	4	5
Cd	ND	ND	ND	ND	ND
Cu	ND	ND	ND	ND	ND
Mn	73.1	39.6	311.4	49.8	74.3
Pb	ND	ND	ND	ND	ND
Zn	63.6	31.0	51.4	26.5	31.7

Roots

Element (mg/kg DW)	1	2	3	4	5
Cd	ND	ND	ND	ND	ND
Cu	54.8	21.7	52.3	23.7	19.8
Mn	56.4	75.2	76.5	562.1	62.1
Pb	100.5	35.1	57.5	61.2	83.2
Zn	140.6	161.5	151.2	220.5	161.1

ND indicates that the concentration was below detection limit. The numbers from 1 to 5 indicate the replications of *A. japonica* seedlings.

(I) Data set for S2 Fig.: Net photosynthetic rates in *A. japonica*

July 2017

PFD (μmol $m^{-2}s^{-1}$)	Net photosynthetic rates (μmol $m^{-2}s^{-1}$)									
	1	2	3	4	5	6	7	8	9	10
1500	3.76	4.20	4.51	3.99	4.15	5.07	6.26	5.08	4.72	4.83
1000	3.53	4.32	4.66	3.41	3.68	4.89	6.66	6.00	5.22	5.41
750	4.01	4.37	4.96	3.97	3.90	4.86	7.29	6.29	4.69	4.68
500	3.81	3.85	4.59	3.49	3.89	4.58	7.30	5.81	4.75	3.99
300	3.43	3.77	4.32	3.45	4.28	5.04	6.80	5.65	4.35	4.33
200	3.36	3.98	4.12	3.42	3.75	4.12	5.96	5.32	4.14	4.31
100	2.62	3.09	3.46	3.16	3.28	3.02	4.38	3.98	3.63	3.92
50	1.80	1.99	1.82	2.25	2.28	1.70	2.30	2.37	2.20	2.55
30	1.29	1.33	1.49	1.35	1.38	1.68	1.34	1.60	1.32	1.67
15	0.14	0.96	0.43	0.19	0.80	0.65	0.50	0.96	0.35	0.72
8	-0.06	0.07	-0.08	0.02	0.21	-0.12	-0.20	0.11	0.01	-0.44
0	-0.15	-0.16	-0.46	-0.28	-0.14	-0.28	-0.70	-0.35	-0.62	-0.67

December 2017-Febrary 2018

PFD (μmol $m^{-2}s^{-1}$)	Net photosynthetic rates (μmol $m^{-2}s^{-1}$)									
	1	2	3	4	5	6	7	8	9	10
1500	3.11	3.32	5.45	4.56	5.08	5.41	4.55	3.93	4.44	5.22
1000	2.61	3.52	5.43	4.18	5.51	5.13	4.83	4.20	4.07	5.94
750	2.77	3.08	5.42	3.26	5.12	5.05	4.79	4.02	3.31	5.31
500	2.29	2.92	4.74	3.96	4.19	4.83	4.93	4.51	3.58	4.78
300	2.66	2.58	4.29	3.54	4.29	4.39	3.76	3.27	3.51	4.33
200	1.47	2.84	3.81	3.04	3.93	3.92	4.32	2.47	3.32	3.48
100	0.63	1.33	3.01	2.90	3.49	2.34	2.30	2.18	2.93	4.44
50	0.85	0.89	1.88	2.10	1.62	1.30	1.39	0.56	1.88	2.76
30	0.37	0.35	1.14	1.71	1.59	0.30	1.08	0.47	1.20	1.34
15	0.32	0.48	0.82	1.02	1.17	0.04	0.33	0.01	1.15	0.61
8	0.17	0.16	0.12	0.30	0.35	-0.23	0.23	-0.25	0.30	0.28
0	-0.29	-0.29	-0.20	-0.34	-0.24	-0.54	-0.60	-0.88	-0.38	-0.16

The numbers from 1 to 10 indicate the replications of *A. japonica*.

(J) Data set for S3 Fig.: Growth rate of annual branch in *A. japonica*.

Replications	Length (mm)									
	July	Aug.	Sept.	Oct.	Nov.	Dec.	Jan.	Mar.	Apr.	May
1	1.0	0.0	0.0	0.1	0.4	0.2	0.1	0.0	12.1	7.9
2	1.2	0.5	0.6	0.2	0.0	0.1	0.2	0.0	12.7	10.4
3	1.4	0.2	0.0	0.2	0.2	0.2	0.2	0.0	15.9	8.3
4	0.5	0.0	0.0	0.3	0.4	0.3	0.2	0.0	10.3	9.5
5	4.5	0.2	0.2	0.5	0.3	0.3	0.1	0.0	12.2	7.3
6	1.0	0.0	0.5	0.7	0.2	0.3	0.0	0.0	5.4	5.4
7	0.5	0.5	0.7	0.2	0.3	0.0	0.1	0.3	9.8	6.9
8	0.2	0.8	0.5	0.0	0.0	0.2	0.2	0.0	15.6	16.3
9	1.0	0.0	2.0	0.0	0.7	0.0	0.3	0.0	5.8	4.1
10	3.0	0.4	0.0	0.4	0.2	0.2	0.0	0.0	17.1	15.8

The numbers from 1 to 10 indicate the replications of *A. japonica*.
