# Supplementary material for: Zn tolerance in the evergreen shrub, Aucuba japonica, naturally growing at a mine site: Cell wall immobilization, aucubin production, and Zn adsorption on fungal mycelia
Source: PLoS One. 2021 Sep 30;16(9):e0257690. doi: 10.1371/journal.pone.0257690 (PMC8483361; doi:10.1371/journal.pone.0257690)
Supplement: S1 Fig — (PDF) [file pone.0257690.s003.pdf]

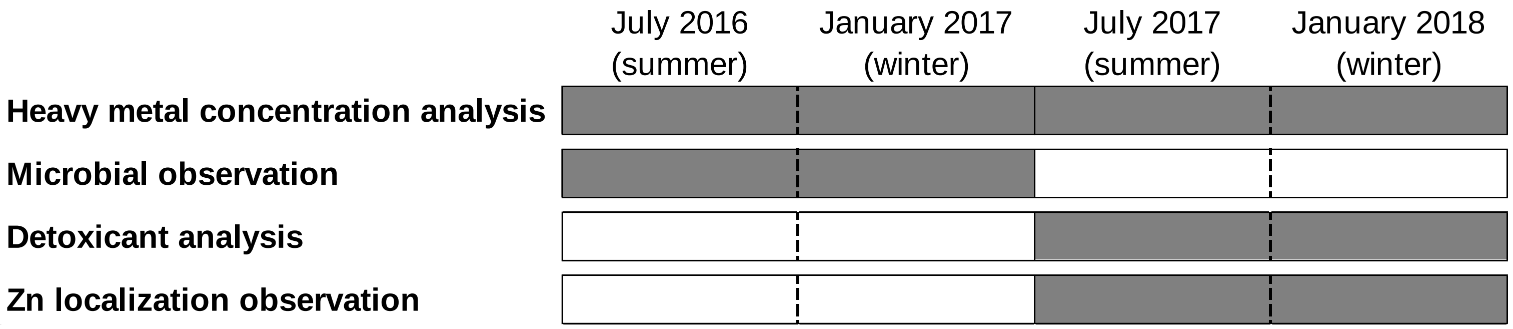
July 2016 (summer)
January 2017 (winter)
July 2017 (summer)
January 2018 (winter)
Heavy metal concentration analysis
Microbial observation
Detoxicant analysis
Zn localization observation
