# Supplementary figures and images for: Zn tolerance in the evergreen shrub, Aucuba japonica, naturally growing at a mine site: Cell wall immobilization, aucubin production, and Zn adsorption on fungal mycelia
Source: PLoS One. 2021 Sep 30;16(9):e0257690. doi: 10.1371/journal.pone.0257690 (PMC8483361; doi:10.1371/journal.pone.0257690)

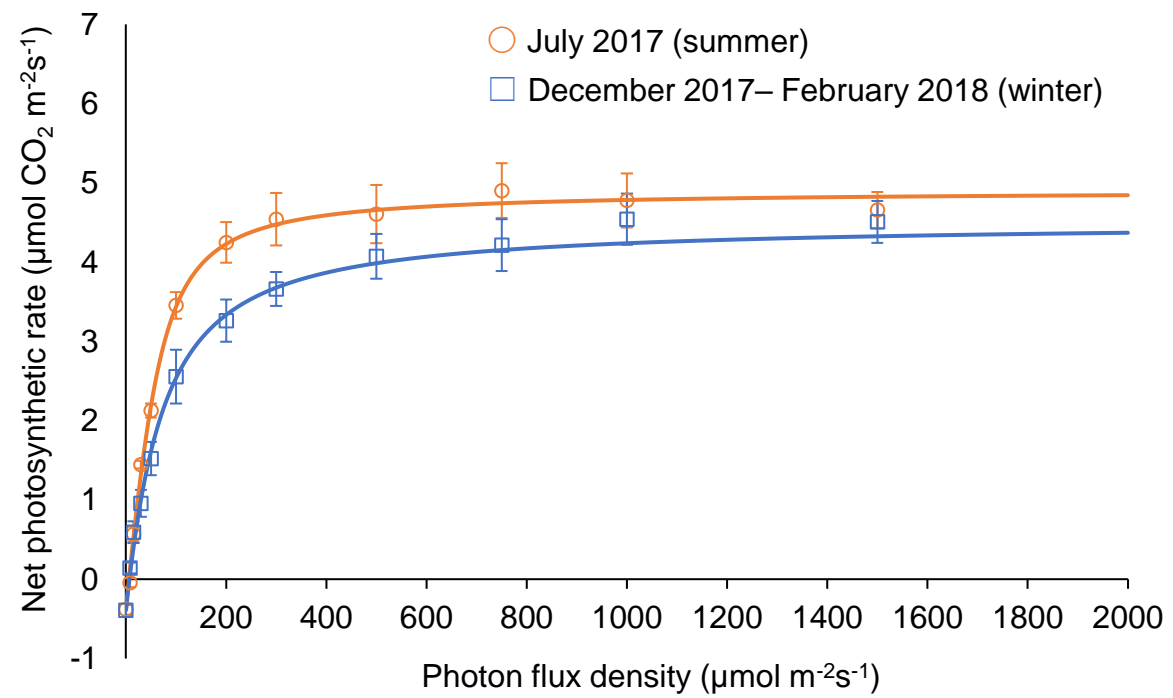

Supplement: S2 Fig — The net photosynthetic rates are shown as the mean ± standard error (n = 10). (PDF) [file pone.0257690.s004.pdf]

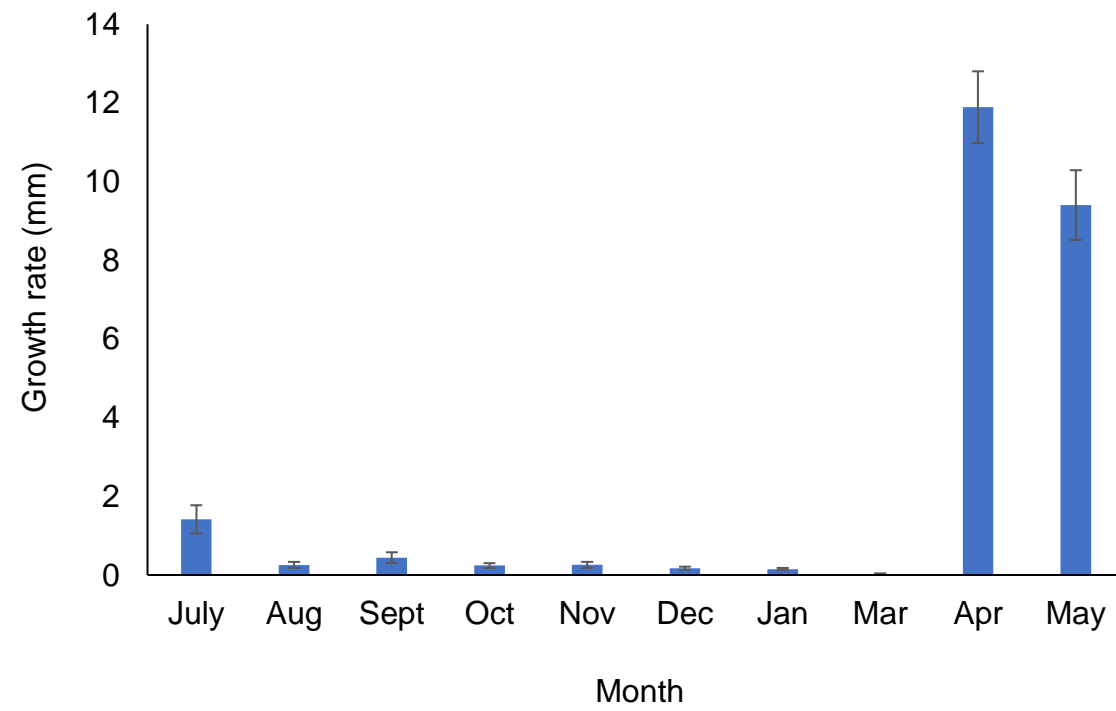

Supplement: S3 Fig — Three branches were arbitrarily selected from each A. japonica (n = 10), and the growth rates (mm) of the annual branches were measured every month from July 2016 to January 2017 and from March 2017 to May 2017. The growth rates were expressed as means ± standard error (n = 10). (PDF) [file pone.0257690.s005.pdf]
