# Supplementary material for: Zn tolerance in the evergreen shrub, Aucuba japonica, naturally growing at a mine site: Cell wall immobilization, aucubin production, and Zn adsorption on fungal mycelia
Source: PLoS One. 2021 Sep 30;16(9):e0257690. doi: 10.1371/journal.pone.0257690 (PMC8483361; doi:10.1371/journal.pone.0257690)
Supplement: S1 Table — Concentrations of heavy metals, exchangeable heavy metals, and pH (H2O) are shown as the mean ± standard error (n = 10). ND indicates that the concentration was below the detection limit. (PDF) [file pone.0257690.s006.pdf]

**Properties**	**Value**
Total Cd (mg/kg)	3.2 ± 0.1
Total Cu (mg/kg)	345.60 ± 12.7
Total Mn (mg/kg)	903.40 ± 158.4
Total Pb (mg/kg)	289.40 ± 19
Total Zn (mg/kg)	122.90 ± 11.5
Exchangeable Cd (mg/kg)	ND
Exchangeable Cu (mg/kg)	2.8 ± 0.5
Exchangeable Pb (mg/kg)	100.0 ± 0.1
Exchangeable Zn (mg/kg)	12.7 ± 1.7
pH (H_2O)	4.7 ± 0.1
Cation exchange capacity [cmol (+) /kg]	46.7
Organic C (%)	12.9
Total N (%)	0.8
