# Supplementary material for: Zn tolerance in the evergreen shrub, Aucuba japonica, naturally growing at a mine site: Cell wall immobilization, aucubin production, and Zn adsorption on fungal mycelia
Source: PLoS One. 2021 Sep 30;16(9):e0257690. doi: 10.1371/journal.pone.0257690 (PMC8483361; doi:10.1371/journal.pone.0257690)
Supplement: S2 Table — Cd, Cu, Mn, Pb, and Zn concentrations are shown as means ± standard error (current-year and 1-year-old leaves in July 2016 and January 2017, n = 6; the other parts, n = 10). Differences were evaluated by Wilcoxon signed-rank test: *, P < 0.05; **, P < 0.01. ND indicates that the concentration was below the detection limit. (PDF) [file pone.0257690.s007.pdf]

| **Element (mg/kg DW)** | **July 2016 (summer)** | **January 2017 (winter)** | ***P* value** | **July 2017 (summer)** | **January 2018 (winter)** | ***P* value** |
|---|---|---|---|---|---|---|
| (a) Current-year leaves | | | | | | |
| Cd | ND | ND | | ND | ND | |
| Cu | 9.9±0.7 | 18.5±4.4* | 0.028 | 9.0±0.6 | 9.4±1.1 | 0.959 |
| Mn | 554.3±83.9 | 839.8±178.1* | 0.046 | 460.1±38.6 | 631.2±44.3* | 0.022 |
| Pb | ND | ND | | ND | ND | |
| Zn | 30.2±2.7 | 24.4±3.4 | 0.249 | 32.1±1.5 | 24.0±1.7 | 0.249 |
| (b) 1-year-old leaves | | | | | | |
| Cd | ND | ND | | ND | ND | |
| Cu | 8.2±2.0 | 12.9±3.0 | 0.116 | 7.7±0.4 | 8.8±0.9 | 0.386 |
| Mn | 1319.5±219.3 | 1345.0±326.7 | 0.753 | 1342.1±143.5** | 990.1±92.4 | 0.007 |
| Pb | ND | ND | | ND | ND | |
| Zn | 32.2±3.6* | 19.8±3.2 | 0.028 | 32.8±1.7* | 28.4±1.7 | 0.047 |
| (c) Branches | | | | | | |
| Cd | ND | ND | | ND | ND | |
| Cu | 51.2±19.8 | 19.5±2.5 | 0.093 | 35.0±5.0* | 19.1±1.4 | 0.022 |
| Mn | 350.0±32.4 | 397.9±27.5 | 0.114 | 286.1±26.1 | 244.6±20.6 | 0.386 |
| Pb | ND | 4.2±0.7 | | ND | ND | |
| Zn | 54.4±6.7 | 49.8±11.7 | 0.575 | 45.7±5.8 | 40.1±5.4 | 0.445 |
| (d) Roots | | | | | | |
| Cd | 29.6±3.9 | 38.5±2.7 | 0.093 | 25.5±4.1 | 28.0±5.0 | 0.721 |
| Cu | 74.3±6.8 | 83.8±10.5 | 0.333 | 100.6±13.3 | 88.6±9.9 | 0.203 |
| Mn | 204.6±19.7 | 292.8±29.4* | 0.022 | 186.3±34.0 | 145.9±14.3 | 0.285 |
| Pb | 220.3±41.9 | 338.0±73.5 | 0.114 | 245.2±75.2 | 170.1±35.9 | 0.285 |
| Zn | 735.9±88.2 | 914.4±65.9 | 0.074 | 669.9±77.2 | 751.9±133.5 | 0.959 |
