# Supplementary material for: Zn tolerance in the evergreen shrub, Aucuba japonica, naturally growing at a mine site: Cell wall immobilization, aucubin production, and Zn adsorption on fungal mycelia
Source: PLoS One. 2021 Sep 30;16(9):e0257690. doi: 10.1371/journal.pone.0257690 (PMC8483361; doi:10.1371/journal.pone.0257690)
Supplement: S3 Table — Cd, Cu, Mn, Pb, and Zn concentrations are shown as means ± standard error (collected in April 2019, n = 5). A. japonica seedlings germinated from October to November in 2018. ND indicates that the concentration was below the detection limit. (PDF) [file pone.0257690.s008.pdf]

S3 Table

Element (mg/kg DW)	Leaves	Cotyledons	Hypocotyls	Roots
Cd	ND	ND	ND	ND
Cu	13.7±0.9	ND	ND	34.5±7.8
Mn	286.3±188.4	641.7±334.2	109.6±50.9	166.5±99.0
Pb	ND	ND	ND	67.5±11.2
Zn	46.0±7.5	34.9±3.1	40.9±7.1	167.0±13.9
